# Roles and Mechanisms of Dipeptidyl Peptidase 4 Inhibitors in Vascular Aging

**DOI:** 10.3389/fendo.2021.731273

**Published:** 2021-08-17

**Authors:** Fen Cao, Kun Wu, Yong-Zhi Zhu, Zhong-Wu Bao

**Affiliations:** ^1^Department of Cardiology, Huaihua First People’s Hospital, Huaihua, China; ^2^Department of Neurology, Huaihua First People’s Hospital, Huaihua, China

**Keywords:** DPP4 inhibitors, vascular aging, diseases, endothelial cells, vascular smooth muscle cells, endothelial progenitor cells, mononuclear macrophages

## Abstract

Vascular aging is characterized by alterations in the constitutive properties and biological functions of the blood vessel wall. Endothelial cells (ECs) and vascular smooth muscle cells (VSMCs) are indispensability elements in the inner layer and the medial layer of the blood vessel wall, respectively. Dipeptidyl peptidase-4 (DPP4) inhibitors, as a hypoglycemic agent, play a protective role in reversing vascular aging regardless of their effects in meliorating glycemic control in humans and animal models of type 2 diabetes mellitus (T2DM) through complex cellular mechanisms, including improving EC dysfunction, promoting EC proliferation and migration, alleviating EC senescence, obstructing EC apoptosis, suppressing the proliferation and migration of VSMCs, increasing circulating endothelial progenitor cell (EPC) levels, and preventing the infiltration of mononuclear macrophages. All of these showed that DPP4 inhibitors may exert a positive effect against vascular aging, thereby preventing vascular aging-related diseases. In the current review, we will summarize the cellular mechanism of DPP4 inhibitors regulating vascular aging; moreover, we also intend to compile the roles and the promising therapeutic application of DPP4 inhibitors in vascular aging-related diseases.

## Introduction

Vascular aging is a complex process characterized by a progressive loss of biological integrity and functionality, which increases mortality with advancing age. Accumulating proof illustrated that vascular aging not only augments the independent risk of cardiovascular diseases, including coronary artery disease (CAD), hypertension, heart failure, and dyslipidemia, but also plays a pivotal role in the etiology of common clinical outcomes, such as neurodegenerative diseases, cerebrovascular diseases, and psychological diseases, implying that vascular aging is a serious threat to human health and life.

Dipeptidyl peptidase-4 (DPP4), also known as cell surface antigen CD26, is an omnipresent transmembrane protease that cleaves NH_2_-terminal dipeptides from their abundant substrates, such as glucagon-like peptide-1 (GLP-1), glucagon-dependent insulinotropic polypeptide (GIP), stromal cell-derived factor-1α/C-X-C chemokine receptor type-4 (SDF-1α/CXCR4), and vasoactive peptides and neuropeptides, which are responsible for glucose metabolism, inflammation, vascular function, and immunity. DPP4 inhibitors are a novel available antihyperglycemic drug approved for treating type 2 diabetes mellitus (T2DM) in clinical practice, decreasing DPP4 enzyme activity and further increasing the concentration of DPP4 substrates. Numerous studies concluded that DPP4 expression occurs in the vascular system, which includes endothelial cells (ECs) (1), vascular smooth muscle cells (VSMCs) (2), cardiomyocytes (3), mononuclear cells (4), and many other cell types. There is a belief that DPP4 may help prevent cardiovascular disease through multiple mechanisms.

In recent years, a large number of studies have been conducted to indicate the protective roles of DPP4 inhibitors in the vascular system. Emerging evidences also suggested that DPP4 inhibitors play a causative role in improving vascular function and delaying vascular aging. It has been reported that DPP4 inhibitors exert vascular beneficial roles beyond glycemic control, which degrade the risk for further development of serious comorbidities associated with T2DM, especially chronic vascular complications (5). On one hand, DPP4 inhibitors take part in the control of vascular aging by inhibiting inflammation and oxidative stress at the molecular level. On the other hand, DPP4 inhibitors exert pleiotropic effects on cardiovascular diseases directly through complicated cellular mechanisms, containing improving EC dysfunction, promoting EC proliferation and migration, alleviating EC senescence, obstructing EC apoptosis, suppressing the proliferation and migration of VSMCs, increasing circulating endothelial progenitor cell (EPCs) levels, and preventing the infiltration of mononuclear macrophages. As a consequence, new therapeutic agents for vascular aging are needed to prevent vascular aging-related diseases.

Although manifest progress has been made in the understanding of vascular aging pathogenesis, the underlying interaction between DPP4 inhibitors and vascular aging remains unknown. There has been no review on the role of DPP4 inhibitors and mediated mechanisms in vascular aging. Therefore, this review is to compile the cumulative and current study on DPP4 inhibitors regulating vascular aging, thereby providing theoretical guidance and clinical application for reversing vascular aging-related diseases.

## The Mechanism of DPP4 Inhibitors in Vascular Aging

Vascular aging, an independent risk factor of cardiovascular diseases and an important element in progression to organ aging, is highly related to alterations in the constitutive properties and biological functions of the blood vessel wall (6). Mechanistically, ECs (**Table 1**) and VSMCs (**Table 2**) are involved in the pathogenesis of vascular aging, and structural damage and dysfunction of these cells, such as dysfunction, proliferation, migration, senescence, and apoptosis, are tightly linked to cardiovascular events in humans (26). Indeed, population studies have demonstrated that dysfunction of circulating EPCs (**Table 3**) and mononuclear macrophages (**Table 3**) also plays an important role in vascular aging (37, 38). DPP4 inhibitors exert pleiotropic effects on cardiovascular diseases directly through complicated cellular mechanisms as described above. Herein we discussed the potential mechanism of DPP4 inhibitors in vascular aging at cellular levels.

**Table 1:** DPP4 inhibitors associated with vascular function and mechanisms in ECs aging.

Vascular aging	DPP4 inhibitors	Functions	Mechanisms	References
ECs dysfunction	vildagliptin	attenuate ECs dysfunction	activateTRPV4 and meditates Ca2+ uptake	(7)
	linagliptin	ameliorate ECs dysfunction	decrease oxidative stress	(8)
	saxagliptin	improve ECs dysfunction	inhibit AP-1 and NF-κB pathway	(9)
	anagliptin	against ECs dysfunction	inhibit NLRP3 inflammasome activation	(10)
	sitagliptin	inhibit ECs dysfunction	inhibit TNF-α	(11)
	sitagliptin	restore ECs dysfunction	activate beta-adrenergic receptor	(12)
	linagliptin	restrain ECs dysfunction	prevent the decrease of KL expression	(13)
ECs proliferation and migration	DPP4 inhibitors	increase proliferation and migration of rBMVECs	mediate SIRT1/HIF‐1α/VEGF signaling pathway	(14)
	teneligliptin	increases HUVECs proliferation	administrate cell-cycle inhibitors hallmarks expression (P27, P21 and P53), and decreasing proapoptotic genes (BAX and CASP3)	(15)
	sitagliptin	promote human aortic ECs proliferation	enhance expression of VEGF	(16)
	anagliptin	increase of ECs migration	activate SOD-1/RhoA/MAPK) signaling	(17)
ECs senescence	saxagliptin	attenuate ECs senescence	regulate AMPK /SIRT1/ Nrf2 signaling pathway	(18)
	DPP4 inhibitors	reverse HUVECs senescence	modulate PKA signaling	(19)
ECs apoptosis	sitagliptin	dampen endothelial apoptosis	SDF-1α/CXCR4/Stat3 signaling pathways	(20)

TRPV4, transient receptor potential vanilloid 4; AP-1, activator protein 1; NF-κB, nuclear factor κB; NLRP3, NLR family, pyrin domain containing 3; TNF-α, tumor necrosis factor alpha; KL, klotho; SIRT1, sirtuin 1; VEGF, vascular endothelial growth factor; SOD-1, superoxide dismutase 1; MAPK, mitogen-activated protein kinase; Nrf2, nuclear factor erythroid 2-related factor 2; PKA, protein kinase A; SDF-1α, stromal cell-derived factor-1α; CXCR4, C-X-C chemokine receptor type-4; Stat3, signal transducer and activator of transcription 3.

**Table 2:** DPP4 inhibitors associated with vascular function and mechanisms in VSMCs aging.

Vascular aging	DPP4 inhibitors	Functions	Mechanisms	References
VSMCs proliferation and migration	anagliptin	downregulate the proliferation of VSMCs	restrain ERK phosphorylation	(21)
	linagliptin	weaken VSMCs proliferation	regulate caspase-3-mediated apoptosis of VSMCs	(22)
	gemigliptin	anti-proliferative roles in	activate NRF-2 signaling pathway and inhibit expression of MCP-1 and VCAM-1	(23)
	sitagliptin	ameliorate VSMCs	prevent Akt/MAPK signaling pathway via upregulating PTEN	(24)
	vildagliptin	suppress VSMCs proliferation	activate ER stress/NF-κB pathway	(2)
	linagliptin	moderate VSMCs proliferation	decrease VSMCs DNA synthesis	(25)

ERK, extracellular signal-regulated kinase; NRF-2, nuclear factor erythroid 2-related factor 2; MCP-1, monocyte chemoattractant protein-1; VCAM-1, vascular cell adhesion molecule-1; Akt, v-akt murine thymoma viral oncogene homologue; MAPK, mitogen-activated protein kinase; PTEN, phosphatase and tensin homolog deleted on chromosome ten; ER, endoplasmic reticulum; NF-κB, nuclear factor κB.

**Table 3:** DPP4 inhibitors associated with vascular function and mechanisms in EPCs and mononuclear macrophage aging.

Vascular aging	DPP4 inhibitors	Functions	Mechanisms	References
EPC aging	Sitagliptin	Protect EPC function	Activate AMPK/ULK1 signaling pathway	(27)
	Sitagliptin	Promote EPC mobilization	Increase plasma SDF-1 and GLP-1 level	(28)
	Saxagliptin	Recruits EPCs from bone marrow	SDF-1α/CXCR4 axis	(29)
	Linagliptin	Improve circulating EPC function	Promote CD34/CXCR4 activity	(30)
	Sitagliptin	Increase EPC count	SDF-1α/CXCR4 axis	(31, 32)
Mononuclear and macrophage aging	Teneligliptin	Reduce ox-LDL uptake and foam cell formation	Inhibit the expression of CD36 and ACAT-1 gene	(33)
	Gemigliptin	Inhibit foam cell formation	Akt/AMPK-dependent NF-κB and JNK signaling	(34)
	Anagliptin	Suppress TNF-1α-induced monocyte migration	Increase adenosine receptor signal pathway	(21)
	Sitagliptin	Recruit circulating monocytes	Upregulate the serum levels of MCP-1	(32)
	Anagliptin	Hinder macrophage accumulation	anti-inflammation	(35)
	Trelagliptin	Inhibit monocyte attachment	Inhibit AP-1 and NF-κB signaling	(36)

MAPK, mitogen-activated protein kinase; ULK1, unc-51-like kinase 1; SDF-1α, stromal cell-derived factor-1α; GLP-1, glucagon-like peptide-1; CXCR4, C-X-C chemokine receptor type-4; ACAT-1, acetyl-CoA acetryltransferase 1; Akt, v-akt murine thymoma viral oncogene homologue; NF-κB, nuclear factor κB; JNK, c-Jun N-terminal kinase; MCP-1, monocyte chemoattractant protein-1; AP-1, activator protein 1.

### DPP4 Inhibitors and EC Function

ECs, the inner layer of blood vessels, act as an essential interface between blood and peripheral tissue elements and possess regulating effects on vascular functions including control of vascular permeability, vascular tone, blood coagulation, extracellular matrix remodeling, and inflammatory responses (39, 40). Hyperglycemia is one of the principal factors contributing to the progression of endothelial dysfunction in diabetes mellitus (41). The clinical studies included small numbers of subjects with T2DM, and the results confirmed an improvement in the vascular function by DPP4 inhibitors (42, 43). In this regard, we will discuss the relationship between DPP4 inhibitors and EC functions.

#### DPP4 Inhibitors and EC Dysfunction

EC dysfunction, also called impaired vascular dilatation, is a pivotal initiation and progression of cardiovascular diseases, bringing about organ damage during aging (44, 45). A growing number of *in vivo* and *in vitro* findings suggest that DPP4 inhibitors have a protective effect against EC dysfunction. Gao et al. proved that vildagliptin activates transient receptor potential vanilloid 4 (TRPV4) and then meditates Ca^2+^ uptake, protecting against hyperglycemia-induced EC dysfunction (7). And vildagliptin attenuated EC dysfunction and shortened atherosclerotic lesions in nondiabetic apolipoprotein E-deficient (ApoE−/−) mice, which means that the effects of vildagliptin are independent of the glucose reducing function (46). Linagliptin suppressed the development atherosclerotic lesions in ApoE−/− mice beyond antiglucose action, which is closely relevant to the amelioration of EC dysfunction through decreasing oxidative stress (8). Sitagliptin reduced inflammation-related EC dysfunction and positively ameliorated diastolic dysfunction (DD) in male Dahl salt-sensitive rats (47). It has been recommended that the DPP4 inhibitor saxagliptin improves EC dysfunction induced by oxidized low-density lipoprotein (ox-LDL) *via* regulating activator protein 1 (AP-1) and the nuclear factor kappaB (NF-κB) pathway (9). Moreover, another result identified that anagliptin provides protection against vascular dysfunction in ECs triggered by hyperglycemia, and the action is mediated by the inhibition of NLR family, pyrin domain containing 3 (NLRP3) inflammasome activation that relies on the sirtuin 1 (SIRT1)-dependent pathway (10). Surprisingly, Goncalves et al. displayed that sitagliptin has a positive capacity of inhibiting vascular EC dysfunction induced by the pro-inflammatory cytokine tumor necrosis factor alpha (TNF-α) (11). Oliveira et al. also found that sitagliptin restores EC dysfunction by the activation of the beta-adrenergic receptor *in vivo* and *in vitro* (12). At last, in a clinical study that recruited 40 patients with CAD and uncontrolled T2DM, sitagliptin significantly alleviated EC dysfunction and inflammatory condition, which exhibits potent effects on preventing vascular-related diseases (48).

Klotho (KL), as a longevity gene, is an anti-aging protein against inflammation. KL methylation is associated with accelerated aging (49, 50). The expression of KL in parathyroid, adipocytes, brain, vascular ECs, and kidney has been previously suggested (51–53). There are papers that reported that KL provides a protective role of restraining EC dysfunction (13, 54). Importantly, reduced circulating KL content is strongly related with vascular aging (55). First of all, the DPP4 inhibitor linagliptin significantly prevented the decreased expression of KL in the vessel wall, eventually blocking EC dysfunction and aortic stiffness in female mice on a Western diet in the long term (13). Then, linagliptin improved not only the progression of brain aging but also other various aging phenotypes in KL knockout mice, and these effects were attributed to an increasing nitric oxide (NO) bioavailability in the cerebral vascular system (56). Indeed, linagliptin ameliorated angiotensin II (Ang II) signaling, which decreased the KL levels (57). Consequently, the KL-mediated vasculo-protective effects of linagliptin are considered to be accounted for by both regulating NO signaling, which is the downstream target of KL, and Ang II signaling, which is the upstream target of KL.

It is widely accepted that the advanced glycation end-product (AGE) receptor, RAGE, named for its ability to bind to AGEs, activates different intracellular signaling pathways. AGE–RAGE interaction signals induced EC dysfunction *via* four pathways: nicotinamide adenine dinucleotide phosphate (NADPH) oxidase–(reactive oxygen species) ROS, Janus kinase (JAK-2)-signal transducer and activator of transcription 1 (STAT 1), mitogen-activated protein kinase (MAPK)–extracellular signal-regulated kinase (ERK), and phosphoinositide-3-kinase (PI3K)–v-akt murine thymoma viral oncogene homologue (Akt), and the phosphorylated NF-κB accelerated the gene expression of growth factors, proinflammatory cytokines, oxidative stress, and profibrotic cytokines (58–60), all of which promoted the occurrence of aging-related cardiovascular diseases. Interestingly, linagliptin could suppress the AGE–RAGE–evoked oxidative stress. DPP4 inhibitors might be a novel therapeutic target for vascular aging in patients with T2DM by block AGE–RAGE axis (61, 62).

#### DPP4 Inhibitors and EC Proliferation and Migration

The proliferation of ECs plays a crucial role in accelerating endothelial healing and ameliorating vascular dysfunction (63). According to a previous study, it has been concluded that DPP4 inhibitors offer protection from hypoxia/high glucose induced impairments in the proliferation and migration of rat brain microvascular endothelial cells (rBMVECs), and this action may be mediated by the SIRT1/HIF‐1α/vascular endothelial growth factor (VEGF) signaling pathway (14). Pujadas et al. proved that the DPP4 inhibitor teneligliptin increases human umbilical vein endothelial cell (HUVEC) proliferation exposed to hyperglycemia conditions, administrating cell-cycle inhibitor hallmark expression (P27, P21, and P53) and decreasing proapoptotic genes (BAX and CASP3), while promoting the expression of B-cell lymphoma 2 (BCL2) (15). It has been showed that sitagliptin increases circulating SDF-1α levels (64). Neuhaus et al. reported that SDF-1 promotes human aortic EC proliferation by enhancing the expression of VEGF (16). In animal experiments, the author identified an increased proliferation of ECs *via* the SDF1-CXCR4 axis (65). The clinical trial with sitagliptin individuals with T2DM, which is powered to assess the capacity of this DPP4 inhibitor on SDF-1, demonstrated that sitagliptin remarkably suppresses SDF-1 degradation (66). A recent study described that anagliptin upregulates superoxide dismutase 1 (SOD-1) expression, which, in turn, negatively modulates oxide production and activates SOD-1/RhoA/MAPK signaling, ultimately promoting EC migration (17).

#### DPP4 Inhibitors and EC Senescence

Recent data have shown that EC senescence is an outstanding contributor to vascular aging, which subsequently results in vascular aging-related diseases (67, 68). Chen et al. designed an animal trial that revealed that DPP4 inhibitors attenuate vascular aging and EC senescence through regulating oxidative stress by means of the AMP-activated protein kinase (AMPK)/SIRT1/nuclear factor erythroid 2-related factor 2 (Nrf2) signaling pathway; meanwhile, saxagliptin has been shown to restore vascular aging by decreased senescence associated beta-galactosidase (SA-β-gal) activity and protein expression of p53 and p21. In consistency with *in vivo* studies, knockdown or inhibition of DPP4 reduced H_2_O_2_-induced EC senescence of great significance (18). It is speculated that DPP4 inhibitors may be promising therapeutic targets to lessen EC senescence; thereby, DPP4 inhibitors may be a salutary method to combat vascular aging. Similar results obtained from another animal study performed by Oeseburg et al. showed that DPP4 inhibitors reverse reactive oxygen species-induced HUVEC senescence by modulating protein kinase A (PKA) signaling downstream of the GLP-1 receptor (19).

#### DPP4 Inhibitors and EC Apoptosis

An increased rate of EC apoptosis is responsible for vascular aging, destruction of vessel integrity, and disruption of the endothelial barrier, which is closely associated with various vascular aging-related diseases (69). To examine the roles of DPP4 in high glucose-induced EC autophagy and apoptosis, Zhao et al. utilized small interfering RNA (siRNA) knockdown and detected DPP4 and AMPK at the protein level (70). The results concluded that DPP4 upregulates adenosine AMPK phosphorylation to promote EC apoptosis and autophagy (70). Presumably, DPP4 inhibitors may become dramatic agents against apoptosis and autophagy of ECs that participated in the aging of the vasculature. Interestingly, the DPP4 inhibitor sitagliptin was found to effectively dampen high glucose-induced endothelial apoptosis *via* the activation of AMPK phosphorylation in ECs (71). There is also evidence supporting the fact that DPP4 inhibitors perform a significant prevention of the apoptosis in HUVECs under hypoxic states, and SDF-1α/CXCR4/signal transducer and activator of transcription 3 (Stat3) signaling pathways can explain this effect of DPP4 inhibitors (20).

### DPP4 Inhibitors and VSMC Function

VSMCs, the medial layer of blood vessels and the predominant structural element of the vessel wall, not only modulate vasoconstriction and vasodilation but also control blood stream and blood pressure. Vascular aging engages a variety of pivotal signals that act in concert to produce VSMC phenotypic changes (72). Additionally, the main changes of VSMC phenotypes occur when they are exposed to diverse stimuli, and the conversion is crucial for VSMC proliferation and migration.

#### DPP4 Inhibitors and VSMC Proliferation and Migration

The proliferation of VSMCs that resulted from various stimulations is the predominant reason of the vascular proliferative events. It is widely known that vascular aging is accompanied by arterial stiffness and arteriosclerosis. Overwhelming evidence indicated that DPP4 inhibitors directly play a favorable impact against VSMC proliferation. The outcome showed that the migration of VSMCs from the media to intima and their proliferation under the synthetic condition are responsible for arterial stiffness and arteriosclerosis; in addition to this, soluble DPP4 was proved to enhance cultured VSMC proliferation, and anagliptin was shown to downregulate the proliferation through restraining ERK phosphorylation (21); DPP4 significantly activates the MAPK and NF-κB signaling pathway, leading to the induction of inflammation and proliferation of VSMCs *in vitro* (73). Similarly, the conclusive effect of soluble DPP4 on the proliferation and inflammation of human VSMCs by activating the MAPK and NF-κB signaling involving protease-activated receptor 2 (PAR2) was also presented in another study (74). Subsequently, the observation from a previous animal experiment demonstrated that linagliptin weakens VSMC proliferation after endothelial injury regardless of the glucose control effect, which may be inextricably linked to accessorial caspase-3-mediated apoptosis of VSMCs (22). It has been established that gemigliptin has antiproliferative and antimigratory roles in VSMCs by activating nuclear factor erythroid 2-related factor 2 (NRF-2) signaling pathway and inhibiting the expression of the chemokine monocyte chemoattractant protein-1 (MCP-1) and vascular cell adhesion molecule-1 (VCAM-1) (23). SDF-1α/CXCR4 signaling is a contributor to the occurrences of hypertrophy and proliferation in renal microvascular smooth muscle cells (75). Besides, in cultured human pulmonary arterial smooth muscle cells (PASMCs), the DPP4 inhibitor sitagliptin prevented the Akt/MAPK signaling pathway *via* upregulating phosphatase and tensin homolog deleted on chromosome ten (PTEN) in a dose-dependent manner, leading to the amelioration of the platelet-derived growth factor (PDGF)-BB-induced VSMC proliferation (24). The study with 4 weeks of vildagliptin in diabetic mice suggested that compared with the control group, the stenosis of injured carotid arteries was markedly reduced, and this consequence was achieved *via* suppressing the VSMC proliferation by activation of the endoplasmic reticulum (ER) stress/NF-κB pathway (2). Recently, Takahashi et al. also reported that combined treatment with linagliptin and empagliflozin moderates VSMC proliferation significantly by attractively decreasing VSMC DNA synthesis *in vitro* (25). Taken together, these findings imply that DPP4 inhibitors play a preventative role in the development of the VSMC proliferation procedure against vascular aging.

### DPP4 Inhibitors and EPC Levels

EPCs, defined as CD34+ cells originating from the bone marrow, are major cardiovascular disease risk biomarkers responsible for endothelial repair and neo-angiogenesis and play a vital role in repairing damaged vessels due to vascular aging (37, 76).

A series of both preclinical and clinical dada observed that DPP4 inhibitors are vascular-protective by controlling the function and number of EPCs, which shows striking correlation with vascular diseases and vascular risks (77). First, sitagliptin, as an intervention for protecting EPC function through enhancing autophagy, promotes ischemic angiogenesis by activating the AMPK/unc-51-like kinase 1 (ULK1) signaling pathway (27). The findings suggested that sitagliptin promotes EPC mobilization from the bone marrow to circulation and augments the migration of EPCs from circulation to kidney parenchyma for angiogenesis (78). Sitagliptin presents a tremendous effect in EPC mobilization, differentiation, and homing ascribed to the increase in the plasma SDF-1 and GLP-1 level independent of blood glucose (28). A very recent study has explored that sitagliptin increases mobilization of circulating EPCs and differentiation of early EPCs (79). Second, saxagliptin recruits EPCs from the bone marrow by SDF-1α/CXCR4 to modulate vasculogenesis in a renal ischemia/reperfusion (I/R) model in a dose-dependent manner, showing antioxidant and anti-inflammatory properties to healing renal injury (29). A randomized, single-site, placebo-controlled, double-blind, phase 4 clinical trial found that combination therapy with saxagliptin and metformin exposes a coefficient effect, which improves circulating EPC function, eventually meliorating arterial stiffness, renal function, and systolic blood pressure (80). Furthermore, in subjects with T2DM with established chronic kidney disease (CKD) who received linagliptin in combination with metformin and/or insulin, the randomized controlled trial illustrated a statistically significant improvement of CD34+ EPC migratory function by promoting CD34/CXCR4 activity (30).

Numerous papers investigated the effect of DPP4 inhibitors on the EPC number. The circulating EPC level was closely correlated to the endothelial function and could be thought of as a pathogenic hallmark of vascular complications, whose damage may expedite the progression of diabetic vasculopathy (81). At first, data from a 12-month recent randomized, open-label active-treatment-controlled clinical trial showed that vildagliptin aggrandizes the circulating EPC number (82). Second, the beneficial effects of both alogliptin and gliclazide on circulating EPC levels in T2DM under poor glucose control have been confirmed by a randomized trial (83), the mechanism of which was reasonably mediated by a glucose-lowering effect. Moreover, the improved effects of sitagliptin on endothelial function in participates with T2DM are probably due to an accessorial number of circulating EPCs (84). When compared to patients with T2DM treated with glimepiride, the group that received sitagliptin for 12 weeks showed an obvious increase in the circulating EPC count, and the effect was likely mediated by the SDF-1α/CXCR4 axis (31). The same results were obtained from a small and non-randomized report, sitagliptin exerted a mediating role in increasing the accumulation of SDF-1, which binds to its receptor, and CXCR4 stimulates the mobilization of EPCs from the bone marrow (77). It has been discovered that sitagliptin upregulates the EPC number in T2DM patients modulated by the SDF-1α/CXCR4 signaling pathway (32), which is in line with the result obtained by Aso et al. (31) and Fadini et al. (32). At last, a surprising consequence turned out that saxagliptin and metformin increased the number of EPCs, and there was no significant difference between the two groups (85).

### DPP4 Inhibitors and Mononuclear Macrophage Function

Emerging evidence suggested that the progression of macrophage phagocytosis of extracellular ox-LDL to transform foam cells is one of the early atherosclerotic properties. In obese T2DM mice and patients, teneligliptin has been shown to conspicuously inhibit the expression of CD36 and acetyl-CoA acetryltransferase 1 (ACAT-1) gene, which participate in reducing ox-LDL uptake and foam cell formation of macrophages (33). More recently, the study conducted by Terasaki et al. confirmed that teneligliptin could suppress foam cell formation of macrophages in T1DM by preventing CD36 and ACAT-1 gene expression partially *via* receding the detrimental effects of AGEs (86). NLRP3 inflammasome activation and interleukin-1β (IL-1β) release contribute to the formation of foam cells (87), which is a characteristic of atherosclerosis (AS). Consistently, it is supported that ox-LDL results in IL-1β excretion in human macrophages by adding NLRP3 expression (88). Dai et al. found that DPP4 inhibitors repress NLRP3 inflammasome activation, toll-like receptor 4 (TLR4) signal, and IL-1β secretion in human macrophages *via* declining the activation of protein kinase C (PKC) (89). Foam cell formation requires scavenger receptors (SRs) on macrophages, comprising SRA, CD36, and LOX-1 (90). DPP4 inhibitors regress SRs, CD36, and LOX-1 expression through downregulating PKC activity, which is involved in the formation of foam cells (90). Gemigliptin confers an inhibiting effect on foam cell formation from THP-1 macrophages, and this efficient effect was mediated by attenuating Akt/AMPK-dependent NF-κB and c-Jun N-terminal kinase (JNK) signaling (34). In an animal study, compared with the control or the sodium glucose cotransporter 2 (SGLT2) inhibitor ipragliflozin group, DPP4 inhibitor alogliptin alone or the combination group illustrated further blockage of foam cell formation, ultimately suppressing the development of atherosclerotic plaque substantially (91). The recruitment of monocytes from circulation to the endothelium is a prominent factor in the pathophysiology of the atherosclerotic lesion. An inhibitor of DPP4, anagliptin profoundly suppressed TNF-1α-induced monocyte migration, which resulted from an increasing adenosine receptor signal pathway (21). In ApoE−/− mice, GLP-1 restrained chemokine-induced monocyte migration and macrophage matrix metalloproteinase-9 (MMP-9) levels, which are significant procedures in AS (73). Additionally, DPP4 inhibitors decreased the monocyte number and prevented macrophage infiltration (92). MCP-1 is straightly involved in the recruitment process of circulating monocytes (93). Quite interestingly, in a randomized, placebo-controlled, single-blind, crossover clinical study, sitagliptin reveals anti-atherosclerotic influence through upregulating the serum level of MCP-1 (32). Sitagliptin has been recognized to play an impact on anti-atherosclerosis that is of great significance *via* promoting the alteration into M2 macrophage in plaque and blocking the lipid content of plaque formation; sitagliptin-mediated restraint of AS builds on this M2 polarization through SDF-1/CXCR4 signaling (94). A DPP4 inhibitor anagliptin can adequately hinder the macrophage accumulation plaque area in coronary arteries due to its anti-inflammatory features (35). Moreover, trelagliptin suppressed the adhesion of monocytes to ECs by inhibiting AP-1 and NF-κB signaling, which mediates the process of inflammation and monocyte attachment, accordingly preventing vascular aging-related diseases (36).

## The Role of DPP4 Inhibitors in Vascular Aging-Related Diseases

**Figure 1:**
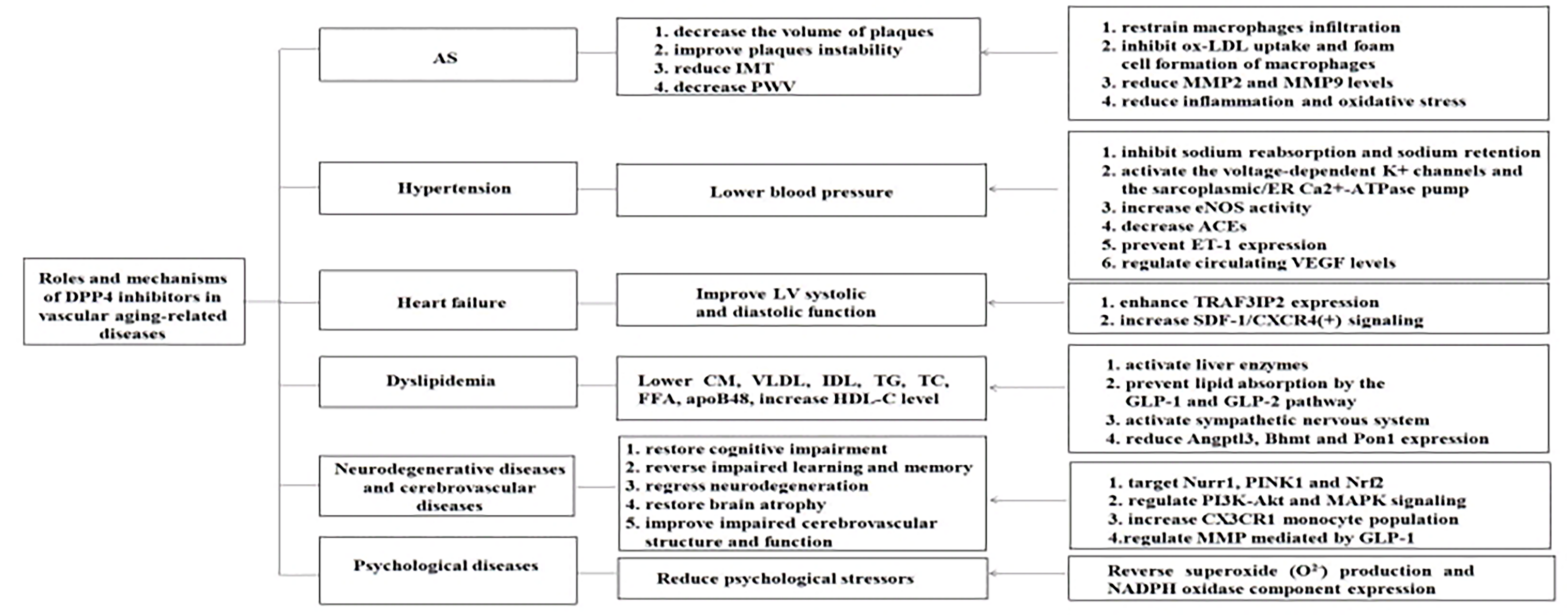
Roles and mechanisms of DPP4 inhibitors in vascular aging -related diseases. DPP4 inhibitors play an effective effect against vascular aging-related diseases through multiple mechanisms. AS, atherosclerosis; JMT, intima-media thickness; PWV, pulse wave velocity; LV, left ventricular; CM, chylomicrons; VLDL, very low density lipoprotein; IDL, intermediate density lipoprotein; TG, triglyceride; TC, total cholesterol; FFA, free fatty acid; apoB48, apolipoprotein B-48; HDL-C, high-density lipoprotein cholesterol; MMP2, macrophage matrix metalloprotcinase-2; MMP9, macrophage matrix metalloproteinasc-9; ER, endoplasmic reticulum; eNOS, endothelial nitric oxide synthase; ACEs, angiotensin-converting enzymes; ET-1, endothelin-1 ; VEGF, vascular endothelial growth factor; TRAF3IP2,TRAF3, Interacting Protein 2; SDF-1, stromal cell-derived factor-Iα; CXCR4, C-X-C chemokine receptor type-4; GLP-1 , glucagon-like peptide-1; GLP-2, glucagon-like peptide-2; Angptl3, angiopoietin-like 3; Bhmt, betaine-homocysteine S-mcthyltransferasc; Pon1, paraoxonasc-1; Nurrl, nuclear receptor related I ; PINK1, PTEN-i nduced putative kinase 1; Nrf2, nuclear factor erythroid 2-related factor 2; PI3K, phosphoinositide-3-kinase; Akt, v-akt murine thymoma viral oncogene homologue; MAPK, mitogen-activated protein kinase; NADPH, nicotinamide adenine dinucleotide phosphate.

**Figure 2:**
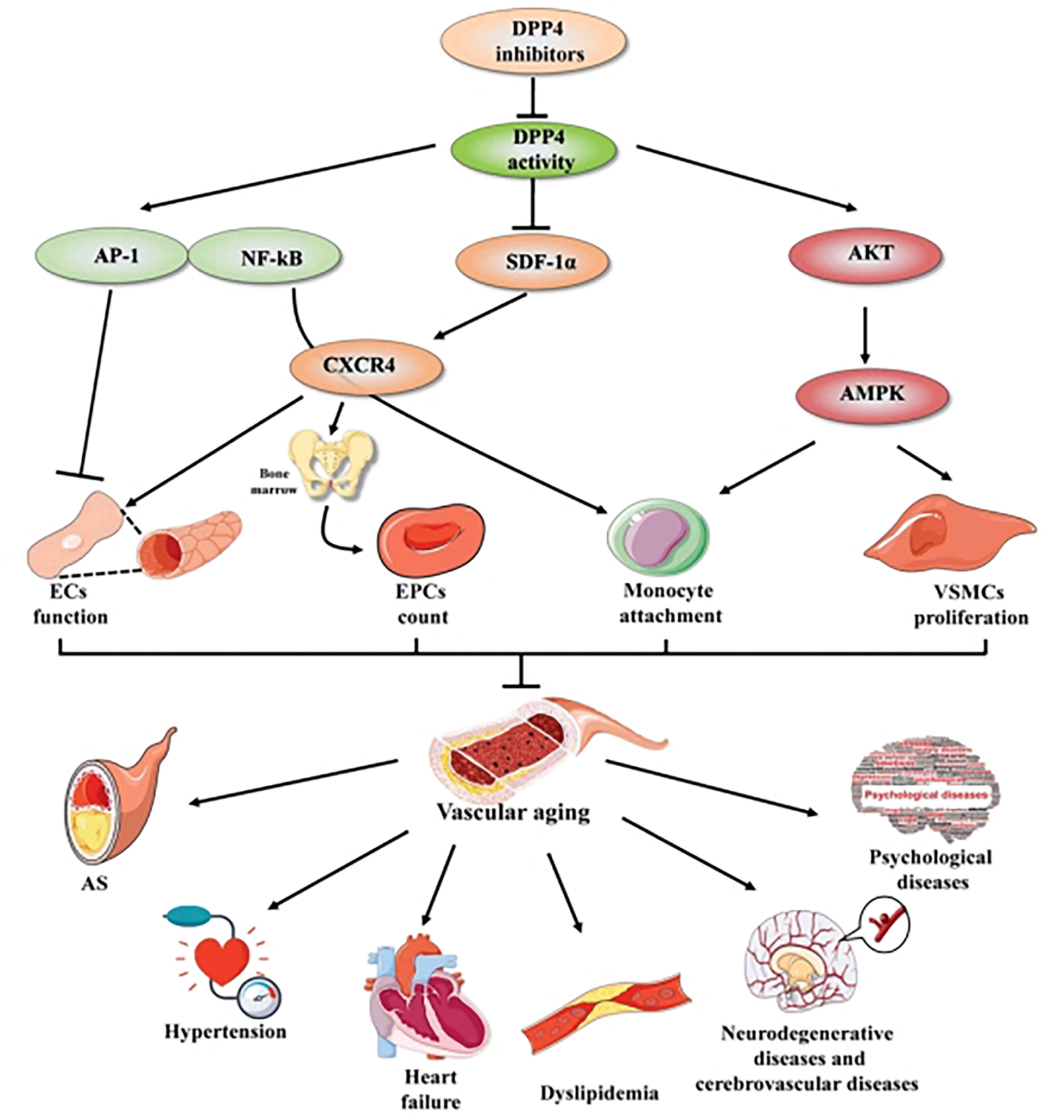
The mechanisms of DPP4 inhibitors in vascular aging and vascular aging-related diseases. DPP4 inhibitors play a beneficial effect in vascular aging not only through multiple cellular mechanisms, including improving ECs function, increasing EPCs count, mediating the process of monocyte attachment and decreasing VSMCs proliferation, but also through complex molecular mechanisms, including AP-I and NF-KB signaling, SDF-1α/CXCR4 axis and Akt/AMPK signaling pathway. Thereby preventing vascular aging-related diseases, compassing AS, hypertension, heart failure, dyslipidemia, neurodegenerative diseases and cerebrovascular diseases, psychological diseases. Arrows indicate stimulatory relationships, indicates inhibitory signal. AP-1, activator protein 1; NF-κB, nuclear factor kappaB; SDF-1, stromal cell- derived factor-1α; CXCR4, C-X-C chemokine receptor type-4; Akt, v-akt murine thymoma viral oncogene homologue; MAPK, mitogen-activated protein kinase; ECs, endothelial cells; VSMCs, vascular smooth muscle cells; EPCs, endothelial progenitor cell; AS, atherosclerosis. Arrows indicate stimulatory relationship, non-arrows indicate inhibitory signal.

The aging of vasculature is a particular type of organic aging that plays a key role in vascular aging-related diseases. Vascular aging-related diseases are the most common complications and the topmost cause of mortality among older people with T2DM; therefore, addressing aging-related vascular diseases is of great significance. Aging-related functional and structural alterations of the vascular wall contribute to the pathogenesis of vascular aging-related diseases, encompassing AS, hypertension, heart failure, dyslipidemia, neurodegenerative diseases, cerebrovascular diseases, and psychological diseases. Recently, successive studies showed that DPP4 inhibitors play a potent effect against vascular aging-related diseases mentioned above (**Figures 1**, **2**).

### DPP4 Inhibitors in AS

AS, as a manifestation of vascular aging and the prominent cause of aging-related cardiovascular disease, is characterized by accumulating lipids and fibrous elements in the vascular wall (95). It is a widespread belief that AS is a complex procedure accompanied by the interaction of diverse cells, lipid, and inflammatory regulators (90). Recently, a growing body of experimental and clinical studies confirmed that DPP4 inhibitors have a favorable role in AS.

In experimental evidences, DPP4 inhibitors substantially restrained macrophage infiltration and markedly decreased the volume of arteriosclerotic plaques in cholesterol-fed rabbits (35). In obese T2DM mice and patients, teneligliptin has been shown to conspicuously inhibit ox-LDL uptake and foam cell formation of macrophages (33). Furthermore, on one hand, sitagliptin negatively modulated the MMP2 and MMP9 levels and reduced plaque MMP9 expression; on the other hand, sitagliptin added amounts of plaque collagen in ApoE knockout mice, which contributes to the stability of arteriosclerotic lesions (73, 92). In addition, combined application with a DPP4 inhibitor and an SGLT2 inhibitor revealed the synergistical suppression of plaque lesion in the aortic root in diabetic mice (91). In consistency with that, teneligliptin inhibited the progression of AS in the aortic arch in ApoE−/− mice that received 20-week drug management by reducing macrophage accumulation, lipid deposition, and MCP-1 expression (96).

DPP4 inhibitors, as anti-atherosclerotic drugs, are being increasingly exploited in clinical research. Firstly, in a prospective, randomized, open-label parallel group trial, DPP4 inhibitors reversed the origination and progression of carotid AS evaluated as intima-media thickness (IMT) in T2DM individuals treated with 12 weeks of sitagliptin and vildagliptin by reducing daily inflammation and oxidative stress. Secondly, a randomized controlled trial, the sitagliptin preventive study of intima-media thickness (IMT) evaluation (SPIKE), demonstrated that sitagliptin has greater reduction in the mean and left maximum IMT in the 104-week treatment group (97). Indeed, the result from a study of preventive effects of alogliptin on diabetic atherosclerosis (SPEAD-A) suggested that alogliptin changes the maximum and mean IMT of carotid arteries, which was estimated by echography (98). In addition, another randomized study analyzed that sitagliptin can regress carotid IMT in patients with T2DM (99), which is consistent with the SPIKE trial. Lastly, aortic pulse wave velocity (PWV) is a hallmark of early AS; decreased PWV was observed in T2DM subjects with 26 weeks of linagliptin treatment (100). However, the PROLOGUE randomized controlled trial concluded that there are no differences on the occurrence of carotid IMT in the sitagliptin administrated group when compared with conventional therapy (101). A further sub-analysis of the PROLOGUE study seems in accordance with this view that sitagliptin has no beneficial effect on the endothelial function and arterial stiffness, which may be conductive to the progression of AS (102–104). Therefore, the roles of DPP4 inhibitors on carotid IMT remain to be investigated. Collectively, DPP4 inhibitors pose potent effects in the management of AS.

### DPP4 Inhibitors in Hypertension

Hypertension is an increasing condition with multiple risk factors, and the occurrence of hypertension is enormous among elderly individuals. Vascular aging, such as arterial stiffness and AS, may be responsible for hypertension in the aging population (105).

A large amount of studies have showed that DPP4 inhibitors are capable of reducing blood pressure levels. A randomized, double-blind, placebo-controlled, three-period, crossover study supported that sitagliptin has a modest antihypertensive function in nondiabetic patients (106). Likewise, it has been documented that sitagliptin has a small reduction in 24-ambulatory blood pressure (BP), which is independent of the glycemic control and body mass index (BMI) (107). Nevertheless, some trials observed that sitagliptin reduces BP significantly (107, 108). In addition, data from a double-blind, randomized, controlled study enrolling 2,000 previously drug-free subjects with T2DM who treated with the drug once or twice daily at a dose of 50 mg for 24 weeks showed that vildagliptin obviously lowered both systolic and diastolic BP (109). Susanne et al. found that using empagliflozin and linagliptin together substantially improved central BP and vascular function compared to a combination of metformin and insulin (110).

The underlying mechanisms for the roles of DPP4 inhibitors on BP are complicated and obscure. First, DPP4 inhibitors enhance the activity of GLP-1, which has been proved to inhibit sodium reabsorption from the proximal tubules and decrease sodium retention, presenting a beneficial impact on BP (111). Second, vildagliptin induced vasorelaxation through the activation of voltage-dependent K+ channels and the sarcoplasmic/ER Ca2^+^-ATPase pump. Third, inhibition of DPP4 increases endothelial nitric oxide synthase (eNOS) activity, causing the NO release and resulting in the dilatation of the vascular wall (112). Fourth, the vascular dilatation induced by acetylcholine and sodium nitroprusside seems to be attributed to the decreased expression of vasoconstrictor-related enzymes afforded by linagliptin, including angiotensin-converting enzymes (ACEs) (113). Additionally, in diabetic rats, sitagliptin prevented the expression of endothelin-1 (ET-1) in the aortic endothelium *via* inhibiting the NF-κB/inhibitor of the NF-κB system by activation of the AMPK pathway, which produces a vascular protection function against hypertension (114). Lastly, vildagliptin performed an antihypertensive action *via* regulating circulating VEGF levels in patients with diabetes and hypertension (115).

### DPP4 Inhibitors in Heart Failure

Vascular aging is a main factor that leads to arterial stiffness, which is responsible for changes in afterload and left ventricular (LV) geometry (116). Numerous lines of proofs from experimental and clinical studies have showed that the favorable properties of DPP4 inhibitors on the progression of heart failure connected with poorer cardiovascular outcomes (117–119), especially DD (120, 121). Alogliptin ameliorated coronary flow reserve (CFR) and left ventricular election fraction (LVEF) assessed by magnetic resonance imaging (MRI) in subjects with T2DM and CAD (122). The same result was achieved by McCormick et al. who found that sitagliptin, another DPP4 inhibitor, improves LV dysfunction in patients with T2DM and CAD during dobutamine stress and a reduction in post-ischemic stunning (123). An animal study showed that use of linagliptin results in the improvement in DD in two rodent models of insulin resistance and obesity. In one study, linagliptin ameliorated DD in insulin-resistant 8-week-old Zucker obese (ZO) rats (124). In another study, linagliptin reversed the development of DD in western diet (WD)−fed mice *via* enhancing the expression of TRAF3 Interacting Protein 2 (TRAF3IP2) and inhibiting oxidant stress and fibrosis (13, 57, 120). DPP4 inhibitors decreased the ratio of transmitral Doppler early filling velocity to tissue Doppler early diastolic mitral annular velocity (E/e’) and increased the ratio of peak early to late diastolic filling velocity (E/A), respectively, in patients with T2DM after acute myocardial infarction (AMI), indicating a beneficial effect on LV diastolic failure (125). The inhibition of DPP4 meliorated cardiac function after AMI by increased SDF-1, which promoted the accumulation of CXCR4(+) bone marrow-derived stem cells into the ischemic area (126, 127). Some studies found that DPP4 inhibitors possess a significant improvement not only in the LV systolic function but also in the diastolic function. For example, patients from a pilot study treated with 12 months of DPP4 inhibitors had an obvious amelioration in systolic, diastolic, and endothelial function (128). In contrast, current evidence from clinical studies has shown that DPP4 inhibitors have no effects on myocardial function in subjects with T2DM and heart failure (129, 130). Data from the EXAMINE trial showed that alogliptin failed to increase the incidence of heart failure outcomes compared with placebo in participants with T2DM after an acute coronary syndrome (131, 132). Same results were obtained from the TECOS trial; sitagliptin has no clinical effect on cardiovascular outcomes (133). Consistently, linagliptin did not increase the risk of heart failure-related outcomes, including among patients with and without a history of heart failure (134). However, some studies proved that DPP4 inhibitors increase the risk of hospital admission for heart failure in those patients with existing risk factors of cardiovascular system or cardiovascular diseases when compared with the control group (135). In SAVOR-TIMI53, saxagliptin was associated with a 27% increased risk of heart failure hospitalization, particularly in the first 12 months (136); the complex mechanisms and potential effects for this have not been fully understood. In consequence, the mechanisms and clinical significance of DPP4 inhibitors on the heart warrant further investigation.

### DPP4 Inhibitors in Dyslipidemia

Existing evidence demonstrated that lipid profiles are highly linked to increased arterial stiffness, which eventually causes vascular aging. Particularly, triglyceride (TG)/high-density lipoprotein cholesterol (HDL-C) ratio, known as the atherogenic index, is a predictor for early vascular aging (137). Population trials have suggested that various DPP4 inhibitors dramatically decrease the level of low-density lipoprotein (LDL) cholesterol, TG, total cholesterol (TC), and free fatty acid (FFA) and add the level of HDL-C both in animal and patient models (138–141). Alogliptin has been showed that it is equipped to downregulate lipids, thereby delaying AS (142). Fukuda-Tsuru et al. found that both single and repeated treatment of teneligliptin lower the level of TG and FFA in plasma under nonfasting states (143). Interestingly, vildagliptin presented a beneficial fasting lipid particle that was related to slight weight loss (109). In participants treated with vildagliptin/metformin, they revealed significantly lower TC and TG and substantially higher HDL-C levels when compared with the glimepiride/metformin treated group (144). Vildagliptin decreased TG and apolipoprotein B-48 (apoB48) in patients treated with a fat rich meal (145). Similarly, evidence from a multicenter, randomized study confirmed that use of anagliptin results in a reduction in the level of fasting apoB48, which is the prominent apolipoprotein of chylomicrons (CM), very low density lipoprotein (VLDL), intermediate density lipoprotein (IDL), and LDL profiles, and the latent mechanism may be attributed to the suppression of the intestinal lipid transport (146). Anagliptin significantly attenuated TC and LDL-C levels, as well as ameliorated glycemic control, especially in female patients (147). Furthermore, sitagliptin downregulated postprandial levels of apoB48-containing lipoproteins, plasma TG, and FFA (148). Observation from a prospective, randomized, multicenter study found that sitagliptin decreased lipid plaque volume in the coronary artery, which was assessed by integrated backscatter (IB)-intravascular ultrasound (IVUS); a marked decrease in the non-HDL cholesterol level may explain this phenomenon (149).

The underlying mechanisms of DPP4 inhibitors in dyslipidemia remain exclusive. First, DPP4 inhibitors lower the secretion of intestinal TG-rich lipoproteins and transform the activation of liver enzymes, which take part in the synthesis and oxidation of lipid (139). Second, DPP4 inhibitors prevent lipid absorption by the GLP-1 and GLP-2 pathway (150). Moreover, DPP4 inhibitors promote postprandial lipid oxidation and mobilization *via* the activation of the sympathetic nervous system (151). Postprandial hypertriglyceridemia exerts a significant role in EC dysfunction and accelerates the development of AS (152). To elaborate the molecular mechanism of lipid lowering impact, the study performed by Zhang et al. found that vildagliptin decreased circulating TC and reversed EC dysfunction in the aorta of diabetic rats that benefited from the reduced expression of angiopoietin-like 3 (Angptl3) and betaine-homocysteine S-methyltransferase (Bhmt) and the elevated activation of paraoxonase-1 (Pon1) (138). Clearly, DPP4 inhibitors are considered to be advantageous for individuals with diabetes and dyslipidemia.

### DPP4 Inhibitors in Neurodegenerative Diseases and Cerebrovascular Diseases

Parkinson’s disease (PD) and Alzheimer’s disease (AD) are age-related neurodegenerative diseases. DPP4 inhibitors are probably novel approaches to the treatment of PD and cognitive impairment by targeting specific pathophysiology proteins, such as nuclear receptor related 1 (Nurr1), PTEN-induced putative kinase 1 (PINK1), and Nrf2 (153). DPP4 inhibitors reversed the impaired learning and memory and regressed AD-like neurodegeneration by GLP-1 signal pathway inclusion of PI3K-Akt and MAPK (154). Recently, saxagliptin revealed a neuroprotective effect on AD, and this effect depends on its functions of anti-inflammatory, antioxidant, antiapoptotic, and neuroprotective mechanisms (155). Linagliptin presented neuroprotective properties in T2DM patients with neurodegenerative disorders through increasing CX3CR1 monocyte population (156). In a diabetic db/db mice model, linagliptin has also been suggested to restore impaired cognitive function and brain atrophy caused by transient cerebral ischemia (157). There is also evidence that that DPP4 inhibitors, mainly including linagliptin, appear to have a potentially favorable impact on improving impaired cerebrovascular structure and function (158, 159). One of the possibly detailed mechanisms is the regulation of MMP mediated by GLP-1 (160, 161). Hence, the DPP4 inhibitor is a conceivable consideration for therapy of neurodegenerative diseases and cerebrovascular diseases.

### DPP4 Inhibitors in Psychological Diseases

Psychological stress can be thought of as a risk factor for vascular aging and vascular aging-related diseases, and oxidative stress plays an important role in vascular senescence in humans and animals (162). Data from the INTERHEART study that recruited 11,119 cases and 13,648 controls from 51 countries illustrated that chronic psychological stressors including depression and anxiety, perception of stress, low sense of control, and life events increase the occurrence of acute myocardial infarction noticeably (163). Indeed, it has been well documented that chronic psychological stressors can increase the likelihood of cardiovascular diseases, hypertension, diabetes mellitus, and metabolic syndrome (164–166). However, some trials demonstrated that diverse stress is harmful to plasma and tissue DPP4 levels (167). Specifically, DPP4 inhibition by anagliptin not only increased the infiltration of macrophage and the expressions of inflammatory molecules, reversing vascular aging and AS, but also reversed superoxide $\left(O_{2}^{-}\right)$ production and NADPH oxidase component expression in ApoE knockout mice (167). Thereby, DPP4 inhibitors can be considered as potential targets against psychological diseases, delaying vascular aging under stress conditions.

## Conclusions

The alternations in the physiological integrity and functionality of blood vessel wall contribute to the aging of vasculature, eventually causing vascular aging-related diseases. DPP4 inhibitors, as a novel antihyperglycemic drug, can efficaciously reduce blood glucose levels. In addition, DPP4 inhibitors also exert pleiotropic impacts in delaying vascular aging beyond glycemic control. The potential mechanisms in this regard at cellular levels are complicated, including improving EC dysfunction, promoting EC proliferation and migration, alleviating EC senescence, obstructing EC apoptosis, increasing circulating EPC levels, suppressing the proliferation and migration of VSMCs, and preventing the infiltration of mononuclear macrophages, all of which showed that DPP4 inhibitors may exert a positive effect against vascular aging, thereby preventing vascular aging-related diseases. In conclusion, DPP4 inhibitors are promising therapeutic targets in the administration of vascular aging and vascular aging-related diseases. However, the safety and efficacy of DPP4 inhibitors have not been fully explored; the mechanisms and clinical significance of DPP4 inhibitors on preventing and treating vascular aging and vascular aging-related diseases warrant further investigation, and further experimental and clinical trial is required.

## Author Contributions

FC wrote the manuscript. KW and Y-ZZ collected the literature, drew the figures, and supervised the manuscript. Z-WB conceived the idea and had been involved in manuscript conception and drafting. All authors read and approved the final manuscript. All authors contributed to the article and approved the submitted version.

## Conflict of Interest

The authors declare that the research was conducted in the absence of any commercial or financial relationships that could be construed as a potential conflict of interest.

## Publisher’s Note

All claims expressed in this article are solely those of the authors and do not necessarily represent those of their affiliated organizations, or those of the publisher, the editors and the reviewers. Any product that may be evaluated in this article, or claim that may be made by its manufacturer, is not guaranteed or endorsed by the publisher.
